# Primary emotions as predictors for fear of COVID-19 in former inpatients with Major Depressive Disorder and healthy control participants

**DOI:** 10.1186/s12888-021-03677-2

**Published:** 2022-02-08

**Authors:** Simon Sanwald, Katharina Widenhorn-Müller, GenEmo Research Group, Maximilian Gahr, Thomas Kammer, Carlos Schönfeldt-Lecuona, Christian Montag, Markus Kiefer

**Affiliations:** 1 Department of Psychiatry and Psychotherapy III, Ulm University, Ulm, Germany; 2 Department of Molecular Psychology, Institute of Psychology and Education, Ulm University, Ulm, Germany

**Keywords:** COVID-19, Corona, Depression, Fear, Stress, Emotion regulation, Primary emotions

## Abstract

**Introduction:**

There are reports of an increase in depressive symptoms and fear during the COVID-19 pandemic, in particular in patients with depression. This study investigates factors related to fear of COVID-19 in former inpatients suffering from depression and healthy controls by assessing variables typically associated with depression and anxiety disorders, i.e. stressful life events (SLEs), the primary emotions SADNESS, PLAY and SEEKING as well as dysfunctional emotion regulation strategies with respect to suppression and reappraisal.

**Methods:**

Data of *n* = 44 former inpatients suffering from depression and *n* = 49 healthy controls were collected. The study had a longitudinal design with two measurement points. Before the pandemic, SLEs, primary emotions, emotion regulation and depression severity were assessed. During the pandemic, COVID-19 associated stressors and life events, emotion regulation, depression severity and fear of COVID-19 were assessed.

**Results:**

Fear of COVID-19 and depression severity during the pandemic were significantly higher in former inpatients than in healthy controls. Depression diagnosis, SLEs and depression severity before the pandemic were significant positive predictors of fear of COVID-19. The primary emotion PLAY was a significant negative predictor of fear of COVID-19. Depression severity did not change significantly in healthy controls.

**Conclusion:**

The results show that risk factors for depression might be risk factors for high fear of COVID-19. In addition, a playful personality could help preventing mental stress in pandemic situations. Thus, positivity based interventions could counteract elevated fear scores during a pandemic.

**Supplementary Information:**

The online version contains supplementary material available at 10.1186/s12888-021-03677-2.

## Background

In 2019 the severe acute respiratory syndrome coronavirus 2 (SARS-CoV-2) triggered the coronavirus disease 2019 (COVID-19) pandemic. In order to avoid overburdening public health systems, governments took undeniably important and unprecedented measures: Restrictions of travelling, restrictions of freedom of assembly and shutting down whole areas. Although necessary, these restriction led to unintended negative side effects such as rising worries about quarantine, isolation/loneliness in parts of the population and many other uncertainties representing a so far unknown psychological burden for the entire society [1, 2]. In line with this, there are reports of an increase of fear and depressive symptoms during the current pandemic [3, 4] and a number of cross-sectional studies reported elevated prevalence ratios of anxiety and depression [5–9]. In addition, a prospective increase in psychological stress during COVID-19 lockdown in a representative family sample has been shown [1]. Another recent study reported anxiety, worry and depressive symptoms to be relevant factors for the level of psychological distress an individual experiences during the current pandemic [10]. Whether individuals with preexisting depression diagnosis are especially vulnerable to fear of the current pandemic, to the best of our knowledge, has not yet been investigated. This is, however, an important research area since evidence suggests that individuals with preexisting mental health issues are significantly more susceptible to the effects of stressful events like a pandemic. For instances, a study on quarantined individuals showed that after controlling for potentially confounding variables such as sex and age a preexisting mental disorder increased the likelihood of persistent anxiety and anger at 4–6 month after quarantine [11]. In addition, lack of social support and disruption in healthcare services during the current pandemic may be especially burdening for individuals with mental disorders who depend on healthcare services [12]. Furthermore, the high comorbidity between anxiety and depressive disorders [13] points towards a vulnerability to fear of the current pandemic when suffering from depression. There are first indications that patients suffering from affective disorders experience elevated stress levels as compared to patients suffering from other mental disorders [14]. Furthermore, depressive symptoms have been shown to be associated with elevated fear of COVID-19 levels [15]. Therefore, this study examines whether there are differences with respect to fear of COVID-19 when comparing former inpatients suffering from depression to healthy controls. Furthermore, since depression and fear of COVID-19 have been shown to be interrelated, in the present study it was assessed whether factors relevant for depression development predict fear of COVID-19 in both groups. Insights from such research could be relevant for the development of interventions tailored to specific factors associated with psychological burden during a pandemic. The following sections provide a short description of Major Depression and the risk factors for developing depression examined in this study.

### Major Depressive Disorder

Major Depressive Disorder (MDD) being the world’s second leading cause of years with disability is a major burden to affected individuals, their social environment and society [16]. Depression has been consistently found to be associated with traumatic or stressful life events (SLEs) [17, 18] and hypothalamic-pituitary-adrenal (HPA) axis dysregulation [19, 20]. The experience of severe SLEs and SLEs in sensitive periods of brain development has been shown to be associated with stress responsiveness to subsequent stressful events [19, 21]. Therefore, SLEs might be relevant predictors of psychological burden in the current pandemic and associated with fear of COVID-19. Moreover, reports of an increased prevalence of depressive symptoms is one of the most common findings after a disaster [22]. According to the diathesis-stress model of depression, a stressor alone does not cause depression. Only the interplay of stress and an individual’s vulnerability to depression development may cause a depressive episode [23]. Personality factors describing a tendency to experience negative effect but also dysfunctional strategies in regulating negative emotions are well established candidates making an individual vulnerable for depression development [24, 25]. The tendency to experience negative emotions but also dysfunctional emotion regulation strategies have been shown to be associated with fear of COVID-19 [26, 27]. Thus, individuals with a preexisting depression diagnosis could be especially vulnerable for elevated levels of fear of COVID-19. The investigation of primary emotions offers the possibility to assess an individual’s tendency to experience different negative but also positive emotions.

### Primary emotions

Affective Neuroscience Theory (ANT) postulates individual differences in emotionality to represent the oldest part of human personality [28]. There are seven primary emotional systems (positive emotions: SEEKING, CARE, PLAY and LUST; negative emotions: FEAR, SADNESS and ANGER) having their well-documented neural substrate in subcortical brain areas [29]. For research linking human personality to psychopathology, primary emotions provide a more direct biopsychological view than classical language-derived approaches to model personality [28, 30, 31]. Thus, the investigation of primary emotions might shed light on fundamental elements of mammalian personality associated with the development and maintenance of mental disorders [29]. Of note, Montag and Elhai (2020) [32] recently also proposed that Panksepp’s Affective Neuroscience Theory is very helpful to better understand the impact of COVID-19 on children/adolescences’ mental health including their caretakers.

According to Watt and Panksepp’s [33] theory of depression development, there are two primary emotional systems of major importance for depression development: On the one hand, SEEKING is defined as the effort made to alleviate negative emotions or the drive to search for vital resources. On the other hand, SADNESS is best described as the emotional state evoked after separation from a loved one. The aforementioned theory postulates that an initial stressor provoking separation distress (in humans the separation may also be a symbolic one) results in a protest phase characterized by efforts to relieve emotional stress. When the individual has no success in soothing separation distress, an emotional shutdown takes place. Thus, the individual saves vital resources. If separation distress in combination with the emotional shutdown is chronically prolonged, however, it will eventually culminate in depression. In accordance with this theory, previous studies found associations between both, low SEEKING as well as high SADNESS and depression severity [34–36]. In line with this, a recent study found depression to be positively associated with SADNESS and FEAR and negatively associated with SEEKING and PLAY [35]. The model used to predict depression in this study was able to explain 52% of the variance in depressive symptoms [35]. Findings of a positive association between the primary emotion SADNESS and SLEs are also in line with the assumption of events associated with at least a symbolical loss triggering the SADNESS system in individuals suffering from depression [36]. This notion is further supported by a model including SLEs, SEEKING and SADNESS predicting a substantial amount of variance in depression onset [36]. Accordingly, SEEKING and SADNESS but also the primary emotion FEAR are obvious candidates for the prediction of fear of COVID-19 [32]. On the other hand, positivity has been found to be a predictor of happiness during the current pandemic [37] and humor has been shown to be negatively associated with fear of COVID-19 [38]. The primary emotion PLAY is defined as social joy – also of relevance for experiencing fun in adulthood [29] - and has been negatively associated with depressive symptoms [35]. Additionally, a humorous manner has been shown to be inversely related to fear of COVID-19 [38], a highly active PLAY system could therefore represent a resilience factor regarding fear of COVID-19. Accordingly, the PLAY system is postulated to have potential for helping patients in adult psychotherapy to reintegrate troublesome emotional experiences towards more adaptive affective trajectories in a playful way [39].

### Emotion regulation

In line with chronically prolonged separation distress, it has been hypothesized that ineffective emotion regulation strategies are risk factors for depression as well as anxiety disorders [24, 40]. In accordance, maladaptive emotion regulation strategies were reported to be associated with fear of COVID-19 [26]. Thus, emotion regulation strategies associated with depression development could be relevant predictors for fear of COVID-19. Previous studies suggest that two emotion regulation strategies, high suppression and low reappraisal, are associated with current (suppression and reappraisal) as well as remitted (only suppression) depression [41]. Suppression describes the inhibition of emotion expressing behavior or emotional reactions [41–43]. Reappraisal involves changing the interpretation of a situation eventually eliciting an emotion thereby changing the situation’s emotional impact [42]. This strategy is especially important for cognitive behavioral therapy, where patients learn how to interpret situations in ways provoking less negative emotions.

### Research question and hypotheses

In summary, fear together with depressive symptoms seem to have increased during the current pandemic [3, 4, 32]. Accordingly, risk factors for depression development like SLEs [17, 18], high SADNESS and low SEEKING [34], high suppression and low reappraisal [41] but also resilience factors like PLAY [37] are potentially relevant for the prediction of fear of COVID-19 as outlined above. Therefore, in this study, SLEs, primary emotions, emotion regulation and depression severity before the pandemic were assessed in former inpatients (at previous admission to the hospital) as well as in healthy controls. At a second point of measurement during the pandemic data was collected in these participant groups on emotion regulation and depression severity. In addition, COVID-19 associated life events and stressors as well as fear of COVID-19 were assessed. This is the first study investigating the prospective influence of risk factors for depression development on fear of COVID-19 in a case-control design. In the present study, the predictive value of SLEs, primary emotions and emotion regulation strategies for fear of COVID-19 was investigated in depressive inpatients as well as in healthy controls. In addition, the association of these variables with the change in depression severity in mentally healthy individuals was examined. Changes in depressive symptoms in former inpatients cannot be attributed to the current pandemic since this group received treatment after the first assessment of depression severity. The assumption was that former inpatients experienced more SLEs and show higher suppression compared to the control group. Moreover, it was expected that former inpatients have higher SADNESS and FEAR scores. Beyond that, former inpatients were expected to score lower on reappraisal, SEEKING and PLAY at the first point of measurement as compared to healthy controls (hypothesis (H) 1). In addition, former inpatients were assumed to have more concerns due to specific aspects coinciding with the pandemic and more severe depressive symptoms, higher suppression and fear of COVID-19 as well as lower reappraisal than healthy controls during the pandemic (H2). Furthermore, it was hypothesized that depression severity increased in healthy individuals as compared to depression severity before the current pandemic (H3). It was further hypothesized that both groups show an increase in suppression and a decrease in reappraisal during the pandemic (H4). Suppression (during the pandemic) was predicted to be positively associated with fear of COVID-19 and depression severity (H5). Reappraisal (during the pandemic) was predicted to be negatively associated with fear of COVID-19 and depression severity (H6). In addition, it was investigated which combination of variables is best suited for predicting fear of COVID-19 in MDD patients and in healthy controls.

## Methods

### Participants

Former inpatients suffering from MDD (*n* = 44; age: *M* = 42.32 years, *SD* = 13.34; 54.5% (*n* = 24) females) and healthy controls (*n* = 49; age: *M* = 38.46 years, *SD* = 13.95; 63.3% (*n* = 31) females) were recruited from the database of the Ulm Gene Brain Behavior Project (UGBBP). Groups did not differ significantly with respect to age (*t*(87) = − 1.33, *p* = .188, *95% CI* [− 9.62, 1.90], note that a U-test provided similar results; for further detail on age groups, see Supplementary Material Table S1) or with regard to the frequencies of sexes (*χ*^*2*^(1) = 0.73, *p* = .393). With respect to the first point of measurement before the pandemic, data partially overlapped with those of earlier studies, whereas data of the second point of measurement (during the pandemic) was not available previously [17, 18, 34]. For further information on recruitment and data collection at the two measurement points see the *Study Design* section below. All procedures performed in this study were in accordance with the ethical standards of the ethics committee of Ulm University, Ulm, Germany and with the 1964 Helsinki declaration and its later amendments. Informed consent of the participants was obtained after the procedures had been fully explained.

### Study design

The current study had a longitudinal design with two points of measurement. Data was collected once per measurement point and individual. Data was collected at admission to the hospital in the group of inpatients. In the group of healthy controls, an appointment for the interview was made after healthy controls had responded to our postings and advertisement. For the second point of measurement, an online questionnaire was completed. The first period of measurement took place from September 14th of 2015 to February 26th of 2020, i.e. before the WHO called out a pandemic:

The sample of depressed inpatients was recruited at the Department of Psychiatry and Psychotherapy III at Ulm University, Ulm, Germany. Inpatients diagnosed for MDD by a psychiatrist at admission to the hospital using the Structured Clinical Interview for DSM-IV (SCID-I) [44] were asked whether they wanted to participate in our study.

A sample of matched healthy controls was recruited by postings in public areas and online advertisement. The control group underwent a diagnostic interview comprising the Mini-DIPS [45] and SCID-II [44] to exclude participants potentially suffering from any kind of mental illness. An additional exclusion criterion was a lifetime diagnosis of any kind of mental or neurological illness or any kind of past psychiatric inpatient treatment or psychotherapy. Both, inpatients and controls, were administered the Critical Life Events Questionnaire (CLEQ), the Beck Depression Inventory (BDI-II), the Emotion Regulation Questionnaire (ERQ) and the Affective Neuroscience Personality Scales (ANPS); all described below. Sociodemographic variables were assessed with a standardized semi-structured interview based on an in-house questionnaire.

The second period of measurement was from July 14th to September 23rd of 2020:

Former inpatients (*n* = 116) and healthy controls (*n* = 91) who had given us consent to contact them for future studies were asked whether they wanted to participate in the current study. At the second point of measurement, *n* = 46 former inpatients and *n* = 50 healthy controls participated. Participants (both groups) were contacted via E-Mail and answered four online questionnaires. The four questionnaires comprised the BDI-II, the ERQ, the Fear of COVID-19 Scale (FCV19-S) and additional five items covering specific COVID-19 associated life events as well as six items covering specific potential stressors associated with COVID-19. Since they did not complete the questionnaires, *n* = 1 healthy control and *n* = 2 former inpatients were excluded from further analyses. The BDI-II and the ERQ were assessed at both points of measurement, whereas all other variables and instruments were assessed once.

The difference in time (in days) between the two points of measurement was initially included in all longitudinal analyses as a covariate.

### Measures or instruments

#### Critical life events questionnaire (CLEQ)

The CLEQ comprises 60 items concerning 30 potentially traumatic life events (such as natural disaster, man-made disaster or death of a close one). There are two questions each for all 30 events assessing whether participants ever experienced the concerning event and, if so, how traumatic they felt about it on a scale from 1 (not traumatic) to 6 (very traumatic) [46]. The product of the occurrence of each event and the experienced severity were added up, thereby calculating a weighted mean.

#### Affective neuroscience personality scales (ANPS)

The ANPS German version [47] assesses individual tendencies in six primary emotional systems with 110 items. The assessed primary emotions are SEEKING, CARE, PLAY (positive emotionality) and SADNESS, FEAR, ANGER, (negative emotionality). The seventh primary emotion of LUST is not assessed by the ANPS since it may potentially have negative carry over effects on the remaining items. Please note that the ANPS assesses individual differences in primary emotional systems as traits. The items are answered on a four point Likert scale (strongly disagree (1) to strongly agree (4)). Internal consistencies for inpatients were acceptable or good (SEEKING: *α* = .82; CARE: *α* = .87; PLAY: *α* = .84; SADNESS: *α* = .68; FEAR: *α* = .87; ANGER: *α* = .77) as they were for healthy controls (SEEKING: *α* = .71; CARE: *α* = .78; PLAY: *α* = .76; SADNESS: *α* = .58; FEAR: *α* = .86; ANGER: *α* = .85).

#### Emotion regulation questionnaire (ERQ)

The ERQ German version [48] assesses two common emotion regulation strategies: suppression and reappraisal. It comprises ten items that are answered on a seven point Likert scale from (1) strongly disagree to (7) strongly agree. Suppression is measured with four and reappraisal with six items. A mean is calculated as long as all items covering the respective emotion regulation strategies are answered. For inpatients suffering from depression internal consistency was good or excellent (measurement point 1: suppression: *α* = .78, reappraisal: *α* = .84; measurement point 2: suppression: *α* = .76, reappraisal: *α* = .92). For healthy controls, internal consistencies were good (measurement point 1: suppression: *α* = .77, reappraisal: *α* = .84; measurement point 2: suppression: *α* = .76, reappraisal: *α* = .81).

#### Beck depression inventory (BDI-II)

Severity levels of depressive symptoms were assessed using the BDI-II German version [49]. The BDI-II is a self-assessment scale comprising 21 items. For each item ratings between 0 (not at all) and 3 (very intensive) are given depending on the symptom complaint. A sum is calculated by adding up ratings of all items. Thus, a maximum of 63 points can be reached. Internal consistency was excellent in the group of individuals suffering from depression with *α* = .91 for the first point of measurement and *α* = .95 for the second point of measurement. Reliability was good for both points of measurement in healthy controls (measurement point 1: *α* = .77; measurement point 2: *α* = .84).

#### Fear of COVID-19 scale (FCV-19S)

The FCV-19S measures the severity of the fear of COVID-19 [50] comprising 7 items that are answered on a five point Likert scale from (1) strongly disagree to (5) strongly agree. A total is calculated by adding up the scores for each item. Thus, the total score ranges from seven to 35. Higher scores indicate greater fear of COVID-19. Internal consistency was excellent for former inpatients (*α* = .93) and good for healthy controls (*α* = .78). The German version was forth- and back-translated independently by two persons speaking both English and German. The items can be found in the Supplementary Material (Table S2).

#### COVID-19 associated life events and stressors

To control for COVID-19 related life events, participants were asked whether they experienced the following events, which they answered with yes or no: infection with SARS-CoV-2, infection of a close person with SARS-CoV-2, death of a close person due to infection with SARS-CoV-2, short time work due to the pandemic and job loss due to the pandemic. In addition, it was assessed how much participants suffered from certain stressors associated with the pandemic using six items: I am very afraid of infecting others with the new corona virus; I have great financial worries due to the current corona pandemic; I am very worried about losing my job because of the current corona pandemic; social distancing in the context of the current corona pandemic is a great burden for me; the restrictions in the organization of my free time due to the current corona pandemic are a great burden for me; I am currently stressed for reasons that have nothing to do with the corona pandemic. These items were rated on a five point scale from 1 (strongly disagree) to 5 (strongly agree). No score was calculated since the purpose of these items was to investigate which specific aspects of the current pandemic are associated with fear of COVID-19.

### Statistical analysis

Statistical analysis was conducted using *R* [51] with the packages *psych* [52], *MASS* [53] and *ggplot2* [54] as well as *JASP* [55]. A stepwise hierarchical linear regression analysis with fear of COVID-19 as the dependent variable was performed. SLEs, primary emotions, emotion regulation and depression severity as well as their two-way interactions with group were predictors. These predictors were measured before the COVID-19 pandemic. In addition, current age, sex and time difference between measurements as well as their interactions with group were added as covariates. Then, predictors were automatically and iteratively added and removed based on AIC comparisons using the *step* command in *R*. All assumptions for multiple linear regression analysis were satisfactorily met.

Fisher’s exact tests were performed to test whether there are group differences in stressful events associated with the COVID-19 pandemic. Using independent sample Welch’s t-tests, differences between former inpatients and healthy controls were investigated with respect to SLEs, emotion regulation, primary emotions, depression severity, fear of COVID-19 and COVID-19 associated stressors (note that U-tests provided similar results). Repeated measures ANCOVAs were calculated to examine changes in emotion regulation strategies and depression severity controlling for sex, age and time between the two points of measurement (in days). Prerequisites for repeated measures ANCOVA were satisfactorily met. Group was not added as between subjects factor since former inpatients had been treated and dismissed from the hospital, therefore group differences with respect to changes in emotion regulation or depression severity are confounded by treatment effects and do not genuinely represent group differences in reactions to the current pandemic situation. To investigate associations with fear of COVID-19 and depression severity during the pandemic, partial Spearman’s correlation coefficients between fear of COVID-19, depression severity, emotion regulation as well as COVID-19 associated stressors controlling for sex and age (even though groups did not differ with respect to these variables, sex and age could still affect within-group associations) were calculated for each group separately. False discovery rate (FDR) was controlled using Benjamini-Hochberg correction [56]. Statistical significance was determined at *p* < .05. Cohen’s *d* is calculated for an estimation of effect sizes for group comparisons. It can be interpreted according to Cohen’s (1988) criteria [57].

## Results

### Experience of COVID-19 related stressful life events

Only 2.0% (*n* = 1) of the participants of the healthy control group and 4.7% (*n* = 2) of the participants of the group of former inpatients were infected with SARS-CoV-2, hence there was no significant difference in infection frequencies (*95% CI* [0.01, 8.76], *p* = .601). 8.2% (*n* = 4) of the group of healthy controls and 11.6% (*n* = 5) of the group of former inpatients indicated that a close person was infected with SARS-CoV-2 (*95% CI* [0.13, 3.49], *p* = .731). No participant stated to have lost a close person due to COVID-19. 18.4% (*n* = 9) of the group of healthy controls and 11.6% (*n* = 5) of the group of former inpatients were affected by short-time work due to the pandemic (*95% CI* [0.47, 7.25], *p* = .396). 6.1% (*n* = 3) of the group of healthy controls and no former inpatients lost their job due to the pandemic (*95% CI* [0.38, infinity], *p* = .244).

### Group differences

Before the Corona pandemic, inpatients reported that they experienced significantly more SLEs than healthy controls. Additionally, inpatients showed significantly higher suppression and less reappraisal than controls did. As expected, inpatients suffering from MDD scored significantly lower on the primary emotion SEEKING but also on the primary emotion PLAY than controls did. Inpatients as compared to healthy controls had significantly higher scores with respect to FEAR and SADNESS. Descriptive and inferential statistics of group differences before the pandemic can be found in Table 1. Group differences in depression severity before the pandemic are not reported since it is considered trivial that inpatients suffering from depression show higher depression severity than healthy controls.

**Table 1:** Group differences between healthy controls and inpatients before the pandemic (age and sex needed not to be controlled for in these analyses, because these variables differed not significantly between control and MDD group)

	*Control*		*MDD*		*df*	*t*	*p*_*BH*_	*95% CI* for mean difference		*d*
	*n*	*M (SD)*	*n*	*M (SD)*				Lower	Upper	
SLEs	49	8.25(7.13)	44	25.00(23.89)	49.88	−4.48	< .001	−24.27	−9.24	− 0.95
Suppression	49	3.47(1.30)	42	4.63(1.51)	81.41	−3.90	< .001	−1.75	−0.57	−0.83
Reappraisal	49	4.72(1.01)	42	3.61(1.29)	77.22	4.50	< .001	0.62	1.60	0.96
SEEKING	48	2.85(0.32)	43	2.46(0.46)	73.56	4.71	< .001	0.23	0.56	1.00
CARE	48	3.02(0.38)	43	2.95(0.55)	74.17	0.73	.551	−0.13	0.27	0.15
PLAY	48	2.97(0.36)	43	2.27(0.47)	78.34	7.94	< .001	0.52	0.87	1.68
FEAR	48	2.30(0.41)	43	3.21(0.51)	80.12	−9.37	< .001	−1.11	−0.72	−1.98
ANGER	48	2.41(0.43)	44	2.49(0.46)	87.88	−0.88	.508	−0.26	0.10	− 0.18
SADNESS	48	2.24(0.30)	44	2.89(0.42)	77.52	−8.51	< .001	−0.81	− 0.50	−1.79

*Note. p*_*BH*_ refers to *p*-values (two-tailed) controlled for FDR. *SLEs* stressful life events, *MDD* group of former inpatients suffering from Major Depressive Disorder

During the pandemic, former inpatients still showed significantly more severe depressive symptoms than controls. In addition, former inpatients reported to experience significantly more fear of COVID-19 than did healthy controls and showed significantly higher suppression scores. There were no group differences with respect to COVID-19-associated fears such as fear of financial hardship or of unemployment. However, former inpatients reported to have elevated psychological strain due to circumstances not associated with the current pandemic. Descriptive and inferential statistics of group differences during the pandemic can be found in Table 2. Note, that U-tests comparing former inpatients with depression severity below a score of 20 (*n* = 17; 34.7%) to healthy controls revealed only one significant group difference: Former inpatients reported more psychological strain not associated with the current pandemic (*p* = .017).

**Table 2:** Group differences between healthy controls and former inpatients during the pandemic (age and sex needed not to be controlled for in these analyses, because these variables differed not significantly between control and MDD group)

	*Control*		*MDD*		*df*	*t*	*p*_*BH*_	*95% CI* for mean difference		*d*
	*n*	*M (SD)*	*n*	*M (SD)*				Lower	Upper	
Depression severity	49	4.96(4.86)	44	23.68(14.80)	51.31	−8.01	< .001	−23.41	−14.03	−1.70
Fear of COVID-19	49	9.78(3.18)	44	13.27(6.65)	60.19	−3.18	.004	−5.70	−1.30	−0.67
Suppression (t2)	49	3.37(1.35)	44	4.25(1.44)	88.32	−3.03	.005	−1.45	−0.30	−0.63
Reappraisal (t2)	49	4.55(1.10)	44	4.01(1.57)	76.23	1.89	.102	−0.03	1.10	0.40
Fear of infecting others	49	2.37(1.27)	44	2.61(1.40)	87.28	−0.89	.508	− 0.80	0.31	−0.18
Financial hardships	49	1.78(0.99)	44	1.82(1.15)	85.29	−0.19	.894	− 0.49	0.40	−0.04
Fear of unemployment	49	1.74(1.06)	44	1.66(1.03)	90.32	0.35	.809	−0.36	0.51	0.07
Stress due to social distancing	49	2.76(1.20)	44	2.98(1.52)	81.75	−0.78	.549	− 0.79	0.35	−0.16
Stress due to restrictions with respect to leisure time activities	49	2.78(1.18)	44	2.77(1.61)	78.02	0.01	.993	−0.59	0.59	0.00
Psychological strain due to other circumstances	49	1.90(1.25)	44	3.59(1.34)	88.22	−6.30	< .001	−2.23	−1.16	−1.31

*Note. p*_*BH*_ refers to *p*-values (two-tailed) controlled for FDR. *MDD* group of former inpatients suffering from Major Depressive Disorder, *COVID-19* coronavirus disease 2019

### Change in symptoms of depression, suppression and reappraisal

After controlling for sex, age and time between the two points of measurement, there were no significant changes in depression severity, neither for healthy controls (depression severity before the pandemic: *M* = 4.53, *SD* = 4.02, *F*(1,44) = 1.33, *p* = .254) nor for former inpatients (depression severity before the pandemic: *M* = 32.01, *SD* = 11.39, *F*(1,34) = 0.58, *p* = .451). There were no changes in suppression or reappraisal, neither for healthy controls (suppression: *F*(1,44) = 0.24, *p* = .626; reappraisal: *F*(1,44) = 0.16, *p* = .688) nor for former inpatients (suppression: *F*(1,34) = 0.14, *p* = .714; reappraisal: *F*(1,34) = 0.13, *p* = .722).

### Correlation analyses

During the pandemic, in former inpatients fear of COVID-19 was significantly positively associated with the fear of infecting others, financial hardship due to the current pandemic and depression severity during the pandemic. Depression severity during the pandemic on the other hand was significantly positively associated with psychological strain due to other circumstances. Depression severity during the pandemic was significantly negatively associated with the emotion regulation strategy of reappraisal. Correlation coefficients for the hypothesized associations were of small to medium size and had the predicted polarities. Spearman’s correlation coefficients and *p*-values for the group of former inpatients can be found in Table 3.

**Table 3:** Spearman’s correlation coefficients between fear of COVID-19, depression severity and the other variables in former inpatients controlling for age and sex

	Fear of COVID-19	*95% CI* for correlation coefficients		Depression severity	*95% CI* for correlation coefficients	
	*r (p*_*BH*_*)*	Lower	Upper	*r (p*_*BH*_*)*	Lower	Upper
Depression severity	.54(.006)**	.26	.74			
Fear of infecting others with coronavirus	.60(.001)**	.34	.77	.35(.119)	.03	.61
Financial hardships due to pandemic	.51(.011)*	.22	.72	.12(.628)	−.21	.42
Fear of unemployment due to pandemic	.38(.097)	.06	.62	.20(.360)	−.13	.49
Stress due to social distancing	.40(.068)	.09	.64	.29(.194)	−.04	.56
Stress due to restrictions with respect to leisure time activities	.35(.119)	.03	.60	.07(.779)	−.26	.38
Psychological strain due to other circumstances	.21(.350)	−.12	.50	.69(< .001)***	.47	.83
Suppression	.31(.184)	−.02	.57	.21(.350)	−.12	.21
Reappraisal	−.26(.263)	−.54	.07	−.47(.018)*	−.69	−.17

*Note. p*_*BH*_ refers to *p*-values (two-tailed) controlled for FDR. Depression severity, suppression and reappraisal during the pandemic. * *p*_*BH*_ < .05, ** *p*_*BH*_ < .01, *** *p*_*BH*_ < .001. COVID-19: coronavirus disease 2019

In the group of healthy controls, fear of COVID-19 was significantly positively associated with fear of infecting others. Depression severity during the pandemic was significantly positively associated with psychological strain due to restrictions in leisure time activities and other circumstances not associated with COVID-19. There were no other significant associations. Spearman’s correlation coefficients and *p*-values for the group of healthy controls can be found in Table 4.

**Table 4:** Spearman’s correlation coefficients between fear of COVID-19, depression severity and the other variables in healthy controls controlling for age and sex

	Fear of COVID-19	*95% CI* for correlation coefficients		Depression severity	*95% CI* for correlation coefficients	
	*r (p*_*BH*_*)*	Lower	Upper	*r (p*_*BH*_*)*	Lower	Upper
Depression severity	.17(.746)	−.13	.45			
Fear of infecting others	.55(.001)**	.30	.73	.18(.746)	−.12	.46
Financial hardships	−.06(.960)	−.35	.24	−.04(.976)	−.34	.26
Fear of unemployment	.12(.850)	−.18	.40	.26(.469)	−.04	.51
Stress due to social distancing	.16(.794)	−.14	.44	.36(.115)	.07	.60
Stress due to restrictions with respect to leisure time activities	.18(.746)	−.13	.45	.42(.043)*	.14	.64
Psychological strain due to other circumstances	−.06(.960)	−.35	.24	.64(< .001)***	.42	.79
Suppression	.01(.999)	−.29	.31	.27(.442)	−.03	.52
Reappraisal	−.01(.999)	−.31	.28	.01(.999)	−.30	.29

*Note. p*_*BH*_ refers to *p*-values (two-tailed) controlled for FDR. Depression severity, suppression and reappraisal during the pandemic. * *p*_*BH*_ < .05, ** *p*_*BH*_ < .01, *** *p*_*BH*_ < .001. COVID-19: coronavirus disease 2019

For associations between fear of COVID-19 and primary emotions see Supplementary Material (Table S3).

### Prediction of fear of COVID-19

**Fig. 1:**
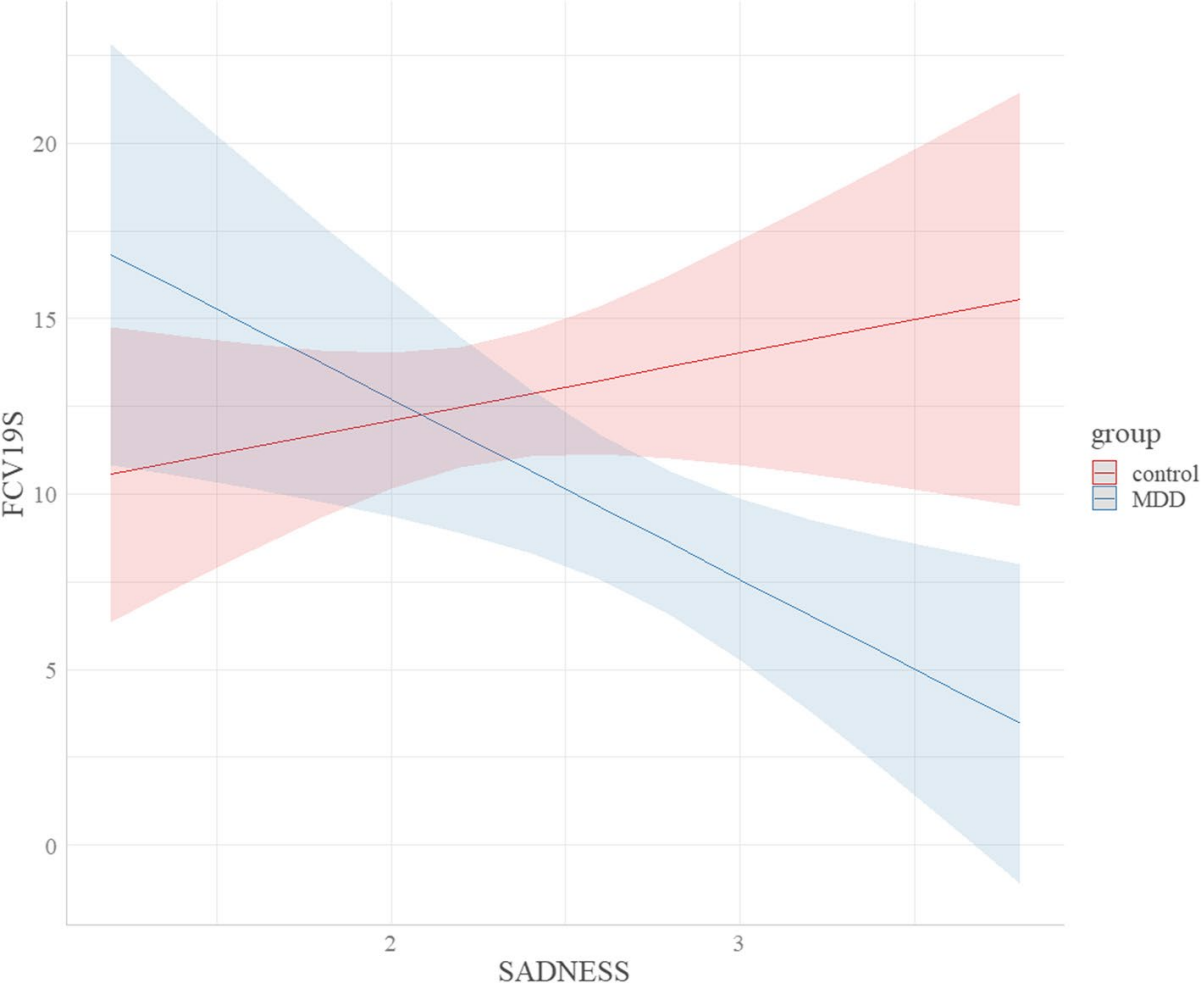
Interaction between group and SADNESS in the prediction of fear of COVID-19

After an automatic stepwise model selection based on AIC as outlined in the *Statistical analysis* section, the model with the lowest AIC explained a significant amount of variance in fear of COVID-19 (*R*^*2*^ = 36.94, *F*(10,72) = 4.22, *p* < .001). Regression coefficients and inferential statistics for the independent variables of the final model can be found in Table 5. The experience of SLEs, a diagnosis of depression and high depression severity before the current pandemic were significantly positively associated with fear of COVID-19. The primary emotion PLAY was significantly negatively associated with fear of COVID-19. Last, there was a significant interaction: While there was a (non-significant) positive association between SADNESS and fear of COVID-19 in healthy controls, SADNESS was negatively associated with fear of COVID-19 in former inpatients (Fig. 1).

**Table 5:** Final model of the stepwise regression analysis with fear of COVID-19 as dependent variable

							*95% CI* for *b*	
Predictor	*b*	*b (std.)*	*SE*	*df*	*t*	*p*	Lower	Upper
(Intercept)	7.92	.23	5.66	72	1.40	.166	−3.36	19.20
Group	15.49	−.37	7.44	72	2.08	.041*	0.67	30.32
Sex	−0.45	.08	1.19	72	−0.38	.705	−2.84	1.93
SLEs	0.09	.39	0.03	72	3.22	.002**	0.03	0.15
PLAY	−2.32	.28	1.16	72	−2.01	.048*	−4.63	−0.02
SADNESS	1.19	−.13	1.86	72	1.03	.306	−1.79	5.63
Reappraisal	0.83	.09	0.55	72	1.50	.138	−0.27	1.93
Depression severity	0.15	.54	0.06	72	2.48	.015*	0.03	0.26
Group*sex	2.70	.15	1.80	72	1.50	.137	−0.88	6.29
Group*SADNESS	−7.06	−.41	2.64	72	−2.67	.009**	−12.33	−1.79
Group*reappraisal	−1.19	−.17	0.74	72	−1.60	.113	−2.66	0.29

*Note.* B (std.) represents standardized coefficients*.* ** *p* < .01, * *p* < .05. *SLEs* stressful life events

## Discussion

In the current study, the predictive value of SLEs, primary emotions and emotion regulation strategies for fear of COVID-19 was investigated in former inpatients with MDD as well as in healthy controls. There were no group differences with respect to the experience of COVID-19 related SLEs. This is worth noting, since group differences in fear of COVID-19 or stress experience related to the current pandemic are unlikely to originate from one group living in a high risk environment for COVID-19 related SLEs. Overall, there were very few controls or inpatients who experienced any of the events in question (please see also that fear of COVID-19 scores were not overly high in both groups). This can be explained by the relatively low rate of infection in Germany in the period from July to September.

The investigation of group differences in variables examined before the current pandemic revealed the expected result pattern, therefore our results support H1: Depressive inpatients reported having experienced more SLEs, show more suppression, less reappraisal, lower SEEKING, lower PLAY, higher FEAR and higher SADNESS than healthy controls did. These findings are in line with previous research [17, 34, 41]. Note that at the first point of measurement, the sample of inpatients suffering from depression partially overlapped with samples analyzed in previous studies [17, 34–36]. SLEs like sexual abuse are associated with an increased risk of a lifetime diagnosis of depression [58]. Depression in turn is associated with a higher reactivity to stressors [59, 60].

Higher stress reactivity could be additionally explained by the use of ineffective emotion regulation strategies, i.e. less reappraisal and more suppression as compared to non-depressed individuals [61, 62]. This is also in line with Watt and Panksepp’s (2009) theory of depression development. They postulate depression to originate from a shutdown mechanism terminating chronically prolonged separation distress [33]. The emotional shutdown is characterized by a tendency to experience less SEEKING and more SADNESS. In sum, SLEs may induce the tendency to experience more emotions that are negative due to subsequent stressors. This in combination with ineffective emotion regulation strategies could lead to an increase in stress experience leaving an individual vulnerable to depression development. It is worth noting, that the current study only provides cross-sectional data and therefore cannot make causal conclusions. However, there is longitudinal data supporting the causal link between stress, negative affect and coping strategies with the development of depressive symptoms [63–65].

Our findings only partially support H2: During the pandemic, former inpatients still showed higher depression severity and a higher use of suppression than did healthy controls. In addition, they experienced more fear of COVID-19 compared to healthy controls. Contrary to our expectations, former inpatients did not experience more concerns due to specific aspects coinciding with the pandemic and reported no significantly lower use of reappraisal.

It seems that former inpatients with still elevated scores of depression severity are more burdened than controls by a fearful view of the pandemic situation, but not by restrictions to limit the incidence of infection. The group difference considering fear of COVID-19 was absent when comparing patients without or with mild depressive symptom complaint to healthy controls. These findings are in line with the results of a recent study reporting more COVID-19 related fear of individuals with mental disorders as compared to healthy participants [66]. Our results extend these findings by showing group differences between healthy individuals and individuals previously diagnosed for depression. Taken together, elevated psychological stress in individuals suffering from depression were related to the perceived threat from the virus. Therefore, in addition to providing facts about the pandemic situation without exaggerating fear, individuals with depression would benefit from a training of effective strategies for dealing with fear of COVID-19. A training of such coping strategies could be, for instance, explicitly incorporated in psychotherapeutic sessions.

In healthy controls, there was no significant change regarding depressive symptoms or emotion regulation strategies. Therefore, our findings do not support H3 and H4. This findings at first glance seem to contradict the many reports of an increase in depressive symptoms [3, 66] or relatively high rates of depression during the pandemic (for a systematic review, see [67]). It is, however, worth noting that the sample of healthy individuals in our control group underwent an extensive screening procedure to exclude individuals with potential signs of mental disorders. Therefore, it is possible that the sample investigated consisted of individuals with a high degree of resilience to stressors associated with the current pandemic. Thus, our results suggest that individuals without indications of mental disorders prior to the pandemic are able to cope with the challenges of a pandemic situation without developing more severe depressive symptoms. Our sample of former inpatients, on the other hand, was assessed before the pandemic when they were in need of an inpatient treatment. After the first point of measurement, they received treatment and at the second point of measurement during the pandemic, they were already dismissed from the hospital. Treatment effects therefore might mask a potential worsening of depressive symptoms in the former inpatient group.

Fear of COVID-19 was significantly positively associated with the fear of infecting others in both groups and with financial hardships as well as with depression severity in former inpatients. This is in line with a recent study reporting perceived risk for loved ones to be related to fear of COVID-19 [68]. The significantly positive association between fear of COVID-19 and depression severity is also in line with recent findings of a positive association between fear of COVID-19 and depression in pregnant wives and their husbands [69]. The fact that the association between depression severity and fear of COVID-19 was present only in former inpatients could reflect the long known comorbidity of depression and anxiety disorders [13]. The positive association between fear of COVID-19 and financial hardships in the group of former inpatients can be explained by depression affecting an individual’s job performance even after symptom improvement [70] in combination with a pessimistic bias in the prediction of future events [71]. Thus, individuals suffering from depression could be in need of economic and social support in times of a pandemic. In this regard, policy-makers should consider the specific needs of patients with depression in the long-term management of the SARS-CoV-2 pandemic.

Contrary to our expectations, there were no associations of fear of COVID-19 with emotion regulation in the control group. In healthy controls, low scores and small variances considering fear of COVID-19 could be responsible for small correlation coefficients in correlational analyses using this variable. It is, however, worth noting that non-significant small to medium size associations in the hypothesized direction between emotion regulation and fear of COVID-19 were found in the group of inpatients.

With respect to depression severity, there was a significantly positive association with psychological strain due to circumstances not associated with the current pandemic in both investigated groups. In addition, reappraisal was significantly negatively associated with depression severity in the group of former inpatients. These findings in combination with the negative association between reappraisal and fear of COVID-19 in the group of former inpatients highlight that depression can be explained by an interaction of vulnerability – resulting from intra-individual (biological and psychological) as well as social interaction factors – and stressful life events. In sum, our findings do not support H5 and partially support H6. The associations between emotion regulation and fear of COVID-19 should be tested in a larger sample of individuals with a diagnosis of depression.

Fear of COVID-19 was predicted by the presence of a diagnosis of depression and higher depression severity. In addition, it is in line with the stable finding of associations between depression and fear as well as anxiety which has been argued to result from common etiologic factors like negative affectivity and neural substrate [72].

PLAY, a primary emotion characterized by a humorous and light-hearted way of dealing with circumstances, was found to be significantly negatively associated with fear of COVID-19 independent of group. This finding supports results of a recent study reporting positivity to have a significant effect on death distress and happiness during the current pandemic [37]. Benign, i.e. self-enhancing humor, has been shown to be effective in down regulating negative and upregulating positive emotions [73]. A playful personality represents a trait of having fun in life and can thus be considered a self-enhancing form of humor. Self-enhancing humor helps in dealing with difficult situations in everyday life [74] and is an important coping strategy in case of SLEs [75]. PLAY is known to be a bottom-up driver of Extraversion [28] and Extraversion itself has been associated with higher life satisfaction in many studies [76]. Taking all this together, even though the effect size is small, this finding is considered to be of major importance since it might help not only healthy but especially mentally burdened individuals dealing with the current pandemic (see Supplementary Material Table S3). In particular in individuals with depression, self-enhancing humor could be explicitly trained in psychotherapeutic sessions in order to improve coping with the pandemic situation.

The association of group and SADNESS is an interesting but unexpected finding. A possible explanation is that SADNESS or separation distress causes a time-limited response to loss in healthy individuals and initially an increase of fear of COVID-19. If, however, SADNESS is transformed into something recurrent because of avoidance behavior, a depressive episode develops [77]. Avoidance behavior is known to reduce fear in short-term but to be responsible for the maintenance of fear in the long-term [78]. Therefore, increased SADNESS associated avoidance behavior in former inpatients may explain the contradictory associations in the comparison of both groups. Nevertheless, and this limits the interpretation, the SADNESS measure of the ANPS is known to be stable and to measure a trait [79].

SLEs were also a factor explaining a significant amount of variance in fear of COVID-19. Research postulating the experience of SLEs to increase an individual’s vulnerability to subsequent stressors and risk of developing a MDD or anxiety disorder [80] provides an explanation for increased fear during a pandemic as a function of SLEs.

Taken together, our study extends previous findings of positive associations between neuroticism and fear of COVID-19 in cross-sectional designs [27, 81] by showing that the primary emotional basis of human personality measured before the pandemic has predictive value for fear of COVID-19 in both healthy controls and former inpatients suffering from depression. The current study showed that FEAR and SADNESS initially differed between former inpatients and healthy controls and an interaction of SADNESS and group was a significant predictor of fear of COVID-19. Since neuroticism is associated with all negative primary emotions [28, 31], our study provides additional specificity as to which emotional facets of neuroticism are associated with fear of COVID-19.

There have been reports showing that “intolerance of uncertainty” can be a predictor of fear of COVID-19 as well as for mental-wellbeing and psychological distress [82, 83]. This is in line with the results of the current study, since it is plausible that SLEs and their emotional processing lead to long-term consequences in terms of an individual’s tolerance for uncertainty, which in turn can result in increased stress and higher levels of fear during a pandemic such as COVID-19. Thus, tolerance of uncertainty could provide additional relevant insights on being vulnerable to future stressors. To make a long story short: the joint investigation of different determinants of fear of COVID-19 like SLEs, primary emotions (here defined as dispositional emotional traits) and tolerance of uncertainty could promote the development of a comprehensive theory of who in particular is at risk to suffer from affective consequences of novel and drastic events like the COVID-19 pandemic.

In terms of practical implications, humor or positivity based interventions could help to prevent the stress level in difficult situations like a pandemic from rising above a critical point. In addition, humor-based online interventions could be especially important in times of a pandemic, being available everywhere at a low threshold while enabling the maintenance of social distance. Such humor-based online interventions have already been shown to effectively increase happiness and reduce depressive symptoms [84]. Therefore, the development of online interventions tailored to pandemic situations could be worthwhile in order to maintain not only the physical but also the mental health of the population.

In addition, interventions with the aim of increasing the public’s knowledge about mental health and the existing infrastructure for getting help in case of mental health issues are important [85]. Furthermore, individuals that suffered from depression showed higher fear of COVID-19. Therefore, by enacting measures to protect against infection, one could simultaneously promote (virtual) interventions that help against depression and would be harmless from the point of view of virus transmission. For instances, online group therapies, and online fitness courses or outside activities with no physical contact where distances can be hold (e.g., work-out programs). Such measures could help building resilience against not only depressive symptoms but also help coping with fear of COVID-19. Since financial hardships are significantly correlated with fear of COVID-19 in former inpatients, it could also be helpful to take measurements promoting financial relief for persons with low income or reduced working capacity due to mental disorders.

Some limitations need to be considered when interpreting the results of our study. First, our sample size was rather small and results should be replicated in a larger sample. Accordingly, it is worth noting that power was too low to detect small effects (for details on post-hoc power analysis, see the Supplementary Material). Therefore, there could be small group differences, changes with respect to depression severity or emotion regulation strategies or within group associations that went unnoticed. Second, the SADNESS scale has relatively low internal consistencies for both groups. Effects regarding SADNESS should therefore be interpreted with caution. Third, our sample underwent an extensive screening procedure ensuring that there was no suspicion of mental disorder in healthy controls and a diagnosis of MDD in the sample of former inpatients was carefully confirmed during the hospital stay before the pandemic. However, participants could have developed mental disorders that went unnoticed during the pandemic. Fourth, the carefully screened sample of healthy participants reduces generalizability of our results to the common population. Fifth, assessment during the pandemic was during the summertime when the infection rate was rather low in Germany. Thus, restrictions were not as strict as they were in spring or in winter. Therefore, the questions examined in our study should be reconsidered in times with high infection rates and major restrictions. Sixth, the time interval between the measurement point before the pandemic and the measurement point during the pandemic considerably varied across individuals. Although we included this time interval as covariate in all longitudinal analyses, we cannot fully exclude that this variable influenced results. Last, we did not perform a priori power analyses. However, we had a predefined sample of inpatients and controls in our database. We contacted all of whom we had the permission to do so (inpatients: *n* = 116; controls: *n* = 91) and tried to collect as many data as possible.

## Conclusion

In summary, our study provides novel findings highlighting that former inpatients suffering from depression are more burdened by the current pandemic than healthy controls. However, humor could be a valuable ally in the development of preventive strategies to combat mental stress in pandemic situations. The development and use of online interventions aiming at increasing humor and happiness tailored to pandemic situations could have beneficial effects on mental health when social distancing is needed to counteract the spread of an infectious disease. SLEs, depression severity, primary emotions and emotion regulation strategies might be valuable predictors of which individuals are at risk of developing high fear levels during a pandemic and provide a theoretical framework of depression development from a psychological perspective.

## Supplementary Information

**Additional file 1: Table S1.** Distribution of healthy controls and former inpatients across different age groups. **Table S2.** Items of the FCV-19S translated to German. **Table S3.** Spearman’s correlation coefficients between fear of COVID-19, depression severity and primary emotions controlling for age, sex and time between measurement points in both groups.

## Data Availability

The datasets used and/or analysed during the current study are available from the corresponding author on reasonable request.

## Abbreviations

WHO: World Health Organization
MDD: Major Depressive Disorder
SLEs: Stressful life events
HPA axis: Hypothalamic-pituitary-adrenal axis
AN: Affective Neuroscience
CLEQ: Critical Life Events Questionnaire
COVID-19: Coronavirus disease 2019
ANPS: Affective Neuroscience Personality Scales
ERQ: Emotion Regulation Questionnaire
BDI: Beck Depression Inventory
FCV-19S: Fear of COVID-19 Scale
SARS-CoV-2: Severe acute respiratory syndrome coronavirus 2
SCID: Structured Clinical Interview for DSM-IV
FDR: False discovery rate
