# Supplementary material for: Primary emotions as predictors for fear of COVID-19 in former inpatients with Major Depressive Disorder and healthy control participants
Source: BMC Psychiatry. 2022 Feb 8;22:94. doi: 10.1186/s12888-021-03677-2 (PMC8822792; doi:10.1186/s12888-021-03677-2)
Supplement: Supplementary file 1 — Additional file 1: Table S1. Distribution of healthy controls and former inpatients across different age groups. Table S2. Items of the FCV-19S translated to German. Table S3. Spearman’s correlation coefficients between fear of COVID-19, depression severity and primary emotions controlling for age, sex and time between measurement points in both groups. [file 12888_2021_3677_MOESM1_ESM.docx]

Primary emotions as predictors for fear of COVID 19 in former inpatients with major depressive disorder and healthy control participants

^1^Simon Sanwald* (M. Sc.), ^1^Katharina Widenhorn-Müller (PhD), GenEmo Research Group, ^2§^Christian Montag (PhD) and ^1§^Markus Kiefer (PhD)

^1^Ulm University, Department of Psychiatry and Psychotherapy III

^2^Ulm University, Department of Molecular Psychology, Institute of Psychology and Education

§ Both authors contributed equally to this work

GenEmo Research Group: Maximilian Gahr, Thomas Kammer, Carlos Schönfeldt-Lecuona

Supplementary Material

**Post-hoc power calculations**

For the independent sample *t*-tests we used false discovery rate (FDR) corrected *p*-values. Thus, only medium to large effects became significant with the smallest effect size of a significant effect being *d* = 0.63. Assuming this effect size, *α* = .05 and sample sizes of *n* = 44 and *n* = 49 a post-hoc power analysis indicated a power of *β* = .91. However, we cannot rule out the possibility that there are small effects we did not detect because of our limited sample size. We would need a sample of *n* = 620 participants to detect small group differences with a power of *β* = .80.

Concerning repeated-measures ANOVAs with *α* = .05, a sample size of *n* = 44, one group and two points of measurement a post-hoc power analysis revealed a power of *β* = .90. However, to detect even small effects with *α* = .05 and *β* = .80, we would need a sample size of *n* = 199. Therefore, we cannot rule out the possibility that there are small effects with respect to changes in depressive symptoms, suppression and reappraisal during the pandemic.

With respect to correlation analyses we calculated FDR-corrected p-values and only medium to large correlation coefficients became significant (according to Cohen’s (1988) criteria). A post-hoc power analysis with the effect size of the smallest correlation being significant (r = .42), α = .05 and n = 49 (this association was found in the control group) indicated a power of β = .94. An additional power analysis indicated that for detecting small effects (r = .10) with α = .05 and β = .80 a sample size of n = 614 is required.

Post-hoc Power calculations for the multiple linear regression:

With an effect size of f2 = .59 an alpha = .05 a sample size of n = 83 (after exclusion of cases with missing values, which is necessary for model reduction) and 10 predictors, a post-hoc power calculation revealed a power of beta= .99.

**Age groups**

Table S1 shows the absolute numbers and precentages of participants in age groups for both groups separately.

**Table S1.**

Distribution of healthy controls and former inpatients across different age groups.

|  | Healthy Controls | | MDD | |
| --- | --- | --- | --- | --- |
| Age group | number | % | number | % |
| 18-20 | 0 | 0.0 | 3 | 6.8 |
| 21-30 | 17 | 34.7 | 6 | 13.6 |
| 31-40 | 13 | 26.5 | 10 | 22.7 |
| 41-50 | 7 | 14.3 | 8 | 18.2 |
| 51-60 | 7 | 14.3 | 15 | 34.1 |
| 61-70 | 4 | 8.2 | 1 | 2.3 |
| 71-80 | 1 | 2.0 | 1 | 2.3 |

*Note.* MDD: Major Depressive Disorder.

**Fear of COVID-19 Scale (FCV-19S) (Ahorsu et al., 2020b) German translation**

**1**: Stimme absolut nicht zu; **2**: Stimme nicht zu; **3**: Neutral; **4**: Stimme zu; **5**: Stimme sehr zu

**Table S2**

Items of the FCV-19S translated to German.

| 01 | Ich fürchte mich besonders stark vor dem neuen Coronavirus (SARS-CoV-2). | 1-2-3-4-5 |
| --- | --- | --- |
| 02 | Es ist mir unangenehm, über das neue Coronavirus nachzudenken. | 1-2-3-4-5 |
| 03 | Ich bekomme feuchte Hände, wenn ich über das neue Coronavirus nachdenke. | 1-2-3-4-5 |
| 04 | Ich habe Angst davor, aufgrund des neuen Coronavirus mein Leben zu verlieren. | 1-2-3-4-5 |
| 05 | Wenn ich die Nachrichten oder Berichte über das neue Coronavirus auf sozialen Medien anschaue, werde ich nervös und ängstlich. | 1-2-3-4-5 |
| 06 | Ich kann nicht schlafen, weil ich mir Sorgen mache, mich mit dem neuen Coronavirus zu infizieren. | 1-2-3-4-5 |
| 07 | Mein Herz rast oder klopft, wenn ich darüber nachdenke, mich mit dem neuen Coronavirus zu infizieren. | 1-2-3-4-5 |

**Correlation analyses between the Affective Neuroscience Personality Scales (ANPS), fear of COVID-19 and BDI-II**

In the group of former inpatients, PLAY was significantly negatively associated with fear of COVID-19 and depression severity during the pandemic. FEAR was significantly positively associated with depression severity during the pandemic.

**Table S3**

Spearman’s correlation coefficients between fear of COVID-19, depression severity and primary emotions controlling for age, sex and time between measurement points in both groups.

| **Former inpatients** | **Fear of COVID-19**  *r(p_BH_)* | **Depression severity**  *r(p_BH_)* |
| --- | --- | --- |
| SEEKING | -0.33(.120) | -0.34(.110) |
| FEAR | 0.13(.614) | 0.51(.011)* |
| CARE | -0.25(.233) | 0.05(.898) |
| ANGER | 0.19(.446) | 0.36(.100) |
| PLAY | -0.42(.035)* | -0.56(.005)** |
| SADNESS | 0.02(.952) | 0.28(.184) |
| **Healthy controls** | **Fear of COVID-19**  *r(p_BH_)* | **Depression severity**  *r(p_BH_)* |
| SEEKING | -0.10(.774) | -0.12(.687) |
| FEAR | 0.16(.571) | 0.32(.164) |
| CARE | -0.04(.900) | -0.03(.905) |
| ANGER | -0.02(.934) | -0.13(.687) |
| PLAY | 0.04(.900) | 0.07(.867) |
| SADNESS | 0.22(.395) | 0.22(.395) |

*Note.* *p_BH_* refers to *p*-values (two-tailed) controlled for FDR. Depression severity during the pandemic. * *p_BH_* < .05, ** *p_BH_* < .01, *** *p_BH_* < .001.
